# Cytosine N4-Methylation via M.Ssp6803II Is Involved in the Regulation of Transcription, Fine- Tuning of DNA Replication and DNA Repair in the Cyanobacterium *Synechocystis* sp. PCC 6803

**DOI:** 10.3389/fmicb.2019.01233

**Published:** 2019-06-05

**Authors:** Katrin Gärtner, Stephan Klähn, Satoru Watanabe, Stefan Mikkat, Ingeborg Scholz, Wolfgang R. Hess, Martin Hagemann

**Affiliations:** ^1^Department of Plant Physiology, University of Rostock, Rostock, Germany; ^2^Faculty of Biology, Genetics & Experimental Bioinformatics, University of Freiburg, Freiburg im Breisgau, Germany; ^3^Department of Solar Materials, Helmholtz-Centre for Environmental Research, Leipzig, Germany; ^4^Department of Bioscience, Tokyo University of Agriculture, Tokyo, Japan; ^5^Core Facility Proteome Analysis, University Medicine Rostock, Rostock, Germany; ^6^Freiburg Institute for Advanced Studies, University of Freiburg, Freiburg im Breisgau, Germany; ^7^Department Life, Light and Matter, University of Rostock, Rostock, Germany

**Keywords:** DNA methylation, DNA methyltransferase, DNA topoisomerase, proteome, restriction analysis, suppressor mutant, transcriptome, UV tolerance

## Abstract

DNA methylation plays a crucial role for gene regulation among eukaryotes, but its regulatory function is less documented in bacteria. In the cyanobacterium *Synechocystis* sp. PCC 6803 five DNA methyltransferases have been identified. Among them, M.Ssp6803II is responsible for the specific methylation of the first cytosine in the frequently occurring motif GGCC, leading to N4-methylcytosine (GG^m4^CC). The mutation of the corresponding gene *sll0729* led to lowered chlorophyll/phycocyanin ratio and slower growth. Transcriptomics only showed altered expression of *sll0470* and *sll1526*, two genes encoding hypothetical proteins. Moreover, prolonged cultivation revealed instability of the initially obtained phenotype. Colonies with normal pigmentation and wild-type-like growth regularly appeared on agar plates. These colonies represent suppressor mutants, because the *sll0729* gene was still completely inactivated and the GGCC sites remained unmethylated. The suppressor strains showed smaller cell size, lowered DNA content per cell, and decreased tolerance against UV compared to wild type. Promoter assays revealed that the transcription of the *sll0470* gene was still stimulated in the suppressor clones. Proteomics identified decreased levels of DNA topoisomerase 4 subunit A in suppressor cells. Collectively, these results indicate that GG^m4^CC methylation is involved in the regulation of gene expression, in the fine-tuning of DNA replication, and DNA repair mechanisms.

## Introduction

DNA modification via methylation occurs in organisms from all domains of life. In prokaryotes, this process results in the appearance of ^m6^A, ^m5^C, and ^m4^C, whereas in eukaryotes the ^m5^C methylation is the predominant process. Eukaryotic DNA methylation plays a crucial role for gene expression regulation and was found to be involved in cell cycle regulation, cell differentiation, and cancer development (D’Urso and Brickner, 2014; Vidalis et al., 2016). Among prokaryotes, DNA methylation is well characterized in the restriction-modification (RM) systems to distinguish host DNA from invading foreign DNA (Tock and Dryden, 2005). In RM systems, cognate restriction endonucleases and DNA methyltransferases are cooperating. In addition, many so-called orphan methyltransferases have been detected in prokaryotic genome sequences, which are not involved in an RM system. Some of these are supposed to play regulatory roles, which are not well-understood among prokaryotes.

The increased application of single molecule real time (SMRT) sequencing not only allowed to determine genome sequences but also permitted to characterize the genome methylation pattern (methylome). These experiments revealed that most prokaryotes have highly diverse methylomes supporting the activity of DNA methyltransferases (Blow et al., 2016). However, the function of these DNA methylation patterns is only known for some cases. Generally, it is assumed that they could play important roles in the regulation of gene expression, DNA replication, repair, and many more (Casadesús, 2016; Mouammine and Collier, 2018). The orphan methyltransferase Dam of *Escherichia coli* that modifies the target sequence GATC to G^m6^ATC has been shown to be involved in DNA repair and replication (Casadesús, 2016), gene expression regulation (Erova et al., 2012), and phase variation (Nou et al., 1995). The regulatory role of DNA methylation by orphan methyltransferases is best understood in the bacterial model system *Caulobacter crescentus*, where DNA methylation is crucial for cell cycle control and many other processes (Fioravanti et al., 2013; Mouammine and Collier, 2018). The combined characterization of the methylome and transcriptome in wild-type and methyltransferase-defective mutant cells revealed that DNA methylation also impacts gene expression patterns in the pathogenic bacteria *Vibrio cholera* (Chao et al., 2015) and *Helicobacter pylori* (Estibariz et al., 2019). In *Borrelia burgdorferi* DNA methylation through the natural RM system also impacts the global transcriptome (Casselli et al., 2018).

Cyanobacteria are photoautotrophic prokaryotes that evolved oxygenic photosynthesis, which later was conveyed into the eukaryotes by endosymbiosis giving raise to the plant kingdom (Ponce-Toledo et al., 2017). They can be found in all photic environments and play an important role in global carbon and nitrogen cycles (Field et al., 1998). Moreover, cyanobacteria are also regarded as attractive hosts for a CO_2_-neutral green biotechnology (Hagemann and Hess, 2018). Recently, we analyzed the methylome of the model cyanobacterium *Synechocystis* sp. PCC 6803 (hereafter *Synechocystis*), which does not express an active RM system. However, five different sites are methylated in the DNA of *Synechocystis* and the cognate methyltransferases were assigned (Scharnagl et al., 1998; Hagemann et al., 2018). Three DNA methyltransferases are encoded on the *Synechocystis* chromosome, M.Ssp6803I responsible for ^m5^CGATCG methylation, M.Ssp6803II for GG^4m^CC, and M.Ssp6803III for the dam-like G^m6^ATC methylation. These three enzymes are well conserved in the cyanobacterial phylum (Hagemann et al., 2018), for example close homologs have been characterized in the filamentous strain *Nostoc* sp. PCC 7120 (Matveyev et al., 2001; Elhai, 2015). There are some hints that the cyanobacterial methylome could be modified in response to certain environmental cues (Walworth et al., 2017; Hu et al., 2018), but the overall role of DNA methylation beyond RM systems has not been studied in cyanobacteria so far.

In our previous study, we reported that the mutation of the gene *sll0729* encoding M.Ssp6803II resulted in the largest alterations of growth and pigmentation among the methyltransferase mutants of *Synechocystis* (Hagemann et al., 2018). The initial phenotype was not stable due to the appearance of suppressor mutants, which were used here to analyze the physiological and regulatory roles of the widespread GGCC methylation among cyanobacteria.

## Materials and Methods

### Strains and Culture Conditions

*Synechocystis* sp. 6803 substrain PCC-M (Trautmann et al., 2012) was used in all experiments and served as wild type (WT). The generation of mutant Δ*sll0729* defective in M.Ssp6803II and the complementation strain Δ*sll0729*+*sll0729* and Δ*sll0729*+*ssl1378* are described by Hagemann et al. (2018). Axenic cells were maintained on agar plates supplemented with BG11 mineral medium (Rippka et al., 1979) at 30°C under constant illumination. The transformants were initially selected on media containing 10 μg ml^–1^ Km (Sigma), while the segregation of clones and cultivation of mutants was performed at 50 μg ml^–1^ Km. During the long-term cultivation of mutant Δ*sll0729*, larger colonies appeared with a pigmentation similar to the WT among the bluish colonies characteristic for mutant Δ*sll0729*. Two clones with WT-like appearance named Δ*sll0729::supp*_1 and Δ*sll0729::supp*_15 were selected for further analysis.

For physiological characterization, axenic cultures of the strains were grown photoautotrophically in BG11 medium either under slight shaking in Erlenmeyer flasks at 50 μmol photons m^–2^ s^–1^ or under aeration with air enriched with CO_2_ [5% (v/v)] in batch cultures at 29°C under continuous illumination of 180 μmol photons m^–2^ s^–1^ (warm light, Osram L58 W32/3). Contamination by heterotrophic bacteria was evaluated by microscopy or spreading of 0.2 ml culture on LB plates. The *E. coli* strains TG1, TOP10, and DH5α were used for routine DNA manipulations. *E. coli* was cultivated in LB medium at 37°C. *Synechocystis* growth was followed by measurements of the optical density at 750 nm (OD_750_). Whole cell absorption spectra were abstained with the UV/VIS spectrophotometer (varian, Cary 50). All growth experiments were repeated at least three times to obtain biological replicates. The Tables and Figures show mean values from representative experiments as specified in the Figure legends.

### UV Stress Experiments

To analyze the UV tolerance, the strains were pre-grown under standard conditions to an OD_730_ of 1.0 and diluted with fresh BG11 medium to an OD_730_ of 0.1. Twenty milliliter of the diluted cell suspensions were incubated in open standard petri dishes toward UV-light (UV-A 9.5 W m^–2^, UV-B 0.6 W m^–2^) using Q-Panel-UVA 340 fluorescent lamps (Q-Panel, Cleveland, OH, United States) in the presence 75 μmol photons m^–2^ s^–1^ PAR (Lumilux Deluxe Daylight L15W/950; OSRAM, Munich, Germany) for 36 h. A second petri dish was covered with a 400 nm cut-off filter foil (Folex PR, Folex, Dreieich, Germany) resulting in total UV-A and UV-B elimination. After the incubation time, aliquots were taken, a dilution series was generated and dropped on BG11-containing agar plates without antibiotics. The agar plates were then incubated under standard conditions for 5 days. As non-exposed control served drop dilution plates with cells taken at the beginning of the experiment.

### DNA Manipulations

The isolation of total DNA from *Synechocystis* was performed as previously described (Hagemann et al., 2018). All other DNA techniques, such as plasmid isolation, transformation of *E. coli*, ligations and restriction analysis (restriction enzymes were obtained from Promega and New England Biolabs) followed standard methods. For the restriction analyses using chromosomal DNA from *Synechocystis*, the restriction endonucleases were used in a 10-fold excess and were incubated for at least 16 h at 37°C to ensure complete digestion. Synthetic primers were deduced from the complete genome sequence of *Synechocystis* (Kaneko et al., 1996) and are listed in the Supplementary Table S1.

### RNA Isolation, Northern-Blotting Experiments and DNA Microarrays

RNA extraction and Northern-hybridization with ^32^P-labeled, single-stranded transcript probes was carried out as previously described (Hein et al., 2013). A high-resolution microarray manufactured by Agilent (Design ID 075764, format 8 × 60 K; slide layout = IS-62976-8-V2) was used for transcriptomic analysis. The array design allows the direct hybridization of total RNA without conversion into cDNA and contains probes for all annotated genes as well as all other transcripts identified in the course of comprehensive RNA sequencing studies (Mitschke et al., 2011; Kopf et al., 2014). Prior to labeling 2 μg of total RNA were treated with Turbo DNase (Invitrogen) according to the manufacturer’s protocol and precipitated with ethanol/sodium acetate. Further details of the labeling and hybridization protocol can be found in Voß and Hess (2014). Raw data were processed with the R package Limma. Median signal intensities were background corrected and quantile normalized. The microarray hybridization was performed with at least two biological replicates for each mutant. The used array design contained technical replicates for each single probe and almost all features were covered by several independent probes. Hence, mean values for all probes of a given feature were used for the final calculation of log_2_ fold changes. For statistical evaluation, i.e., the *P*-value calculation, we used the Benjamini–Hochberg procedure. Further details of data processing and statistical evaluation using the R software were described previously (Georg et al., 2009).

The full datasets for the comparison of the Δ*sll0729* mutant, the complementation strains, and WT as well as of the suppressor mutants with WT are accessible from the GEO database with the accession number GSE126285, respectively.

### Cell Size Estimation and DNA Contents

Cells for microscopic observation were collected after 5 days cultivation. One milliliter sample was diluted by 1:10000. The cell diameter of 100 cells was measured using the light microscope Olympus BX41 under 400 fold magnification. For the FACS analysis, cells were grown under 2% CO_2_ or ambient air conditions for 24 h. The FACS analyses for cell size and DNA content estimation were performed as previously described (Watanabe et al., 2012), with minor modification. Chromosomal DNA was stained with 10 μM SYTOX Green (Invitrogen, Carlsbad, CA, United States). For effective permeation of SYTOX Green, we applied freeze-thaw treatment after glutaraldehyde fixation as described by Watanabe and Yoshikawa (2016). Flow cytometry of 30,000 cells was performed on an Accuri C6 flow cytometer (Becton-Dickinson, Palo Alto, CA, United States).

### Generation and Characterization of Promoter Test Strains

Promoter sequences (150 nt upstream of TSS and the 5′UTR) of *sll0470* and *sll1526* were synthesized by BaseClear. In addition to the WT sequence, one variant was synthesized in which the GGCC site was changed to GCGC. To improve subsequent cloning, the sequence of a *Sac*I site was added to the 5′ ends and of a *Pst*I site to the 3′ ends. Subsequently, the promoter fragments were cloned in front of the *luxAB* reporter genes into the *Synechocystis* promoter probe vector pILA (Kunert et al., 2000).

The promoter-containing pILA-vectors were used to transform *Synechocystis* cells. For each transformation, cells from 10 ml of a *Synechocystis* WT or suppressor clone Δ*sll0729::supp_*15 culture (OD_750_ = 0.5–1.0) were harvested by centrifugation (3,750 rpm, 20°C, 10 min). The pellet was re-suspended in 0.2 ml BG11 medium. After addition of 3 μg plasmid (pILA-vector) the sample was incubated at room temperature for 1 h and then plated on 0.75% BG11 agar plates without antibiotics for the WT and with kanamycin (50 μg ml^–1^) for the suppressor clone Δ*sll0729::supp_*15. Slightly shaded plates were incubated for 1–2 days at 30°C. For subsequent selection, streptomycin (5 μg ml^–1^) was added underneath the agar layer. After 2–3 weeks, single colonies were picked and cultivated on plates with increasing streptomycin concentration until reaching 20 μg ml^–1^.

To determine the activities of P_sll0470_ and P_sll1526_, cells were inoculated in BG11 medium. Liquid cultures were diluted to an OD_750_ of 0.5. After 1 day of growth, cultures were diluted once again to an OD_750_ of 0.5 and used for measurements after another 30 min of shaking at 30°C. For the analysis four biological replicates per strain and three technical replicates per biological sample were employed. Non-transparent 96-well plates (PerkinElmer) were used for analysis. 1.5 μl decanal (Alfa Aesar) was loaded in each well and subsequently 0.1 ml of liquid culture was applied per well. Luminescence was measured in a Victor3^TM^ 1420 Multilabel Counter (PerkinElmer). Promoter activity measurements were repeated with cold-stressed cells. For this purpose, cells were incubated at 15°C for 30 min prior to luciferase measurements. Statistical analysis was performed using the two-tailed unpaired Student’s *t*-test (^∗∗∗^*p* < 0.001).

### Proteomic Methods

Cells were disrupted using Precellys 24 homogenizer (peqLab Biotechnologie GmbH, Erlangen, Germany) in a buffer containing 10 mM Tris/HCl, pH 7.4; 138 mM NaCl; 2.7 mM KCl; 1 mM MgCl_2_. An aliquot of the resulting total protein extract was centrifuged at 22,000 × *g* for 100 min to obtain the membrane-enriched fraction. Total protein extracts and the membrane-enriched fraction of three biological replicates of each investigated strain were digested with trypsin and applied to mass spectrometry as described before by Pade et al. (2017). LC-HDMS^E^ analyses were carried out using a nanoAcquity UPLC system coupled to a Synapt G2-S mass spectrometer (Waters, Manchester, United Kingdom). Label-free protein quantification and expression analysis was performed using Progenesis QI for Proteomics (Non-linear Dynamics, Newcastle upon Tyne, United Kingdom). A detailed description of the experimental procedures is provided in the Supplementary Material. In addition to the suppressor mutant strains Δ*sll0729::supp_*1 and Δ*sll0729::supp_*15, we investigated the proteome of the DNA methyltransferase mutants Δ*slr6095* and Δ*sll8009* (Hagemann et al., 2018) to rule out non-specific DNA methylation effects. Only proteins showing changed abundances with fold change values > 2 and corresponding Anova *P*-values < 0.01 in the two suppressor mutant strains compared with the WT and fold change values > 1.5 compared to other methyltransferase mutants were regarded as specifically linked to the DNA methylation via M.Ssp6803II. The mass spectrometry proteomics data have been deposited to the ProteomeXchange Consortium via the PRIDE (Perez-Riverol et al., 2019) partner repository with the dataset identifiers PXD012698 (10.6019/PXD012698) and PXD012751 (10.6019/PXD012751).

To support the lowered abundance of the topoisomerase 4 subunit A (Sll1941) we performed Western-blot experiments. An amount of 7.5 μg total protein extract from the WT and the mutant Δ*sll0729::supp_*1 was separated by SDS-PAGE. One gel was stained by Coomassie-brilliant-blue to verify equal protein loading, while a second gel was blotted onto a PVDF membrane. The blot was incubated with a peptide antibody against Sll1941. Two Sll1941-specific peptides (DRQERLKALKKELRGLKKK and DSAPEAKQDDLNLAVKPTPK) were synthesized and used for rabbit immunization by Davids Biotechnologie (Regensburg, Germany). Specific antibody binding was visualized by chemiluminescence as described by Pade et al. (2015).

## Results

### Gene Expression Analysis of the Mutant Δ*sll0729*

The mutant Δ*sll0729* defective in M.Ssp6803II is unable to methylate the first cytosine within its recognition sequence, GGCC. There are 38,512 GGCC sites occurring on both strands of the *Synechocystis* chromosome, roughly corresponding to one methylation site every 185 bp. Moreover, the mutant was reported to show clear alterations in growth and pigmentation compared to the WT (Hagemann et al., 2018).

**FIGURE 1:**
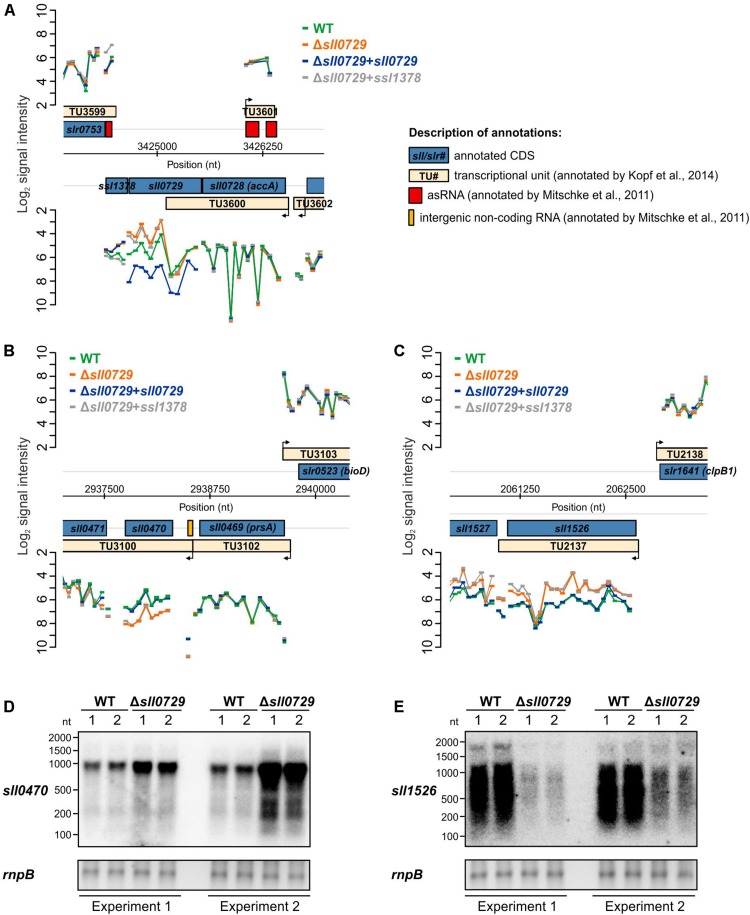
Visualization of the microarray results obtained for specific genomic loci. **(A)** Signal intensities of probes covering the *sll0729* gene on the *Synechocystis* chromosome. **(B,C)** Signal intensities of probes covering the regions harboring *sll0470* and *sll1526*, respectively. **(D,E)** Experimental verification of transcriptional changes in Δ*sll0729* by Northern-blotting experiments using ^32^P-labeled probes specific for *sll0470* **(D)** or *sll1526* **(E)** with RNA obtained from two independent cultivations. In each microarray experiment two biological and three technical replicates were used.

There are reports showing that DNA methylation can be involved in gene expression control in bacteria. Hence, we hypothesized that the missing methylation of GG^m4^CC in the Δ*sll0729* mutant might result in significant expression changes leading to the previously observed phenotypical alterations. To this end, we performed genome-wide microarray experiments for the Δ*sll0729* mutant compared to WT and expected a high number of genes with altered expression due to the high number of GGCC sites. In contrast to our expectations, we only noticed a small number of genes with altered transcription (Supplementary Table S2). Moreover, no clear correlation was found between changed gene expression and the presence of GGCC in close proximity to regulatory sequences (promoters). Thus, some of the observed expression changes might result from pleiotropic effects due to the strong growth and pigment phenotype of Δ*sll0729* reported previously (Hagemann et al., 2018). Therefore, we repeated the transcriptome profiling 3-times using independent experimental setups with different cultivation systems and also including strains in which an intact *sll0729* allele was expressed for complementation (strain Δ*sll0729*+*sll0729*). We further investigated a strain, in which the gene *ssl1378*, which is situated downstream of *sll0729* on the same strand, is ectopically expressed to test for possible polar effects (strain Δ*sll0729*+*ssl1378*). The missing or modified transcription of *sll0729* and *ssl1378* was confirmed by the below-threshold signals in Δ*sll0729* and restored or increased signals for either *sll0729* or *ssl1378* in the respective complementation strains (Figure 1A). Interestingly, we only found the two genes *sll0470* and *sll1526* displaying the expected alterations compared to WT: (i) a significant fold change in Δ*sll0729*, (ii) no change in the strain complemented with *sll0729*, and (iii) a similar fold change as observed in Δ*sll0729* in the strain complemented with *ssl1378* (Table 1 and Figures 1B,C). Moreover, these two genes were the only overlap between four independent DNA microarray experiments using independent RNA isolations from the Δ*sll0729* mutant, which suggests that *sll0729* deletion is indeed responsible for the changed transcript abundances. In addition, it should be noted that the gene *sll1527*, which is located downstream of *sll1526* and probably is co-transcribed with it, shows a similar expression pattern, however, with lower fold changes that did not pass our significance criteria (−0.75 in Δ*sll0729*; −0.93 in Δ*sll0729*+*ssl1378*).

**TABLE 1:** Significant changes in transcript abundances of protein-encoding genes in the Δ*sll0729* mutant compared to WT.

**Gene**	**Δ*sll0729***	**Δ*sll0729+sll0729***	**Δ*sll0729+ssl1378***	**Annotation**
*sll0470*	**1.30**	0.12	**1.30**	hypothetical protein
*sll1526*	**−1.05**	−0.08	**−1.22**	methyltransferase-domain containing protein of unknown function

*Values are given as log_2_ fold changes (FCs) compared to WT and fulfill the set significance criteria, namely a log_2_ FC > 1 or < −1 and a corresponding P-value < 0.05. The table only contains genes with expression changes reproducible in four independent microarray experiments and for which the transcript abundance was restored by ectopic sll0729 expression (strain Δsll0729+sll0729, no significant FC compared to WT) and for which a significant FC was also observed in strain Δsll0729+ssl1378. This strain ectopically expresses the ssl1378 gene, which is most likely co-transcribed with sll0729 and hence, also knocked out in the Δsll0729 mutant. Strain Δsll0729+ssl1378 was used as control, because it exhibited a similar phenotype as Δsll0729, confirming that the here described altered transcript abundances are indeed caused by the mutation of sll0729 and can only be complemented by sll0729 expression. The presented data are mean values from two biological replicates with each three technical replicates from one representative microarray experiment. Numbers in bold show significant expression changes.*

The gene *sll0470* was reproducibly up-regulated, whereas *sll1526* was down-regulated in the Δ*sll0729* mutant compared to WT. To verify the changed transcript abundance for both genes, Northern-blotting experiments were performed using RNA of the WT and Δ*sll0729* from two independent cultivations. Again, in Δ*sll0729* a higher transcript abundance was found for *sll0470*, whereas it was clearly decreased for *sll1526* making these genes promising candidates for direct methylation-dependent effects on their expression (Figures 1D,E).

### Growth and Pigmentation Phenotype of the Δ*sll0729* Mutant Can Be Compensated by Suppressor Mutations

**FIGURE 2:**
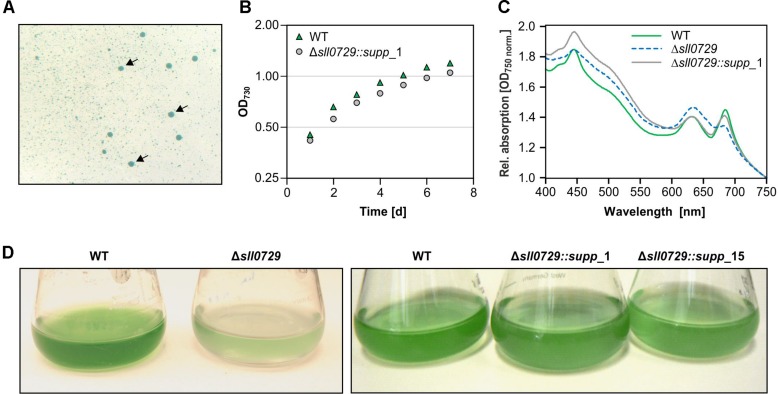
Detection and isolation of suppressor mutant clones Δ*sll0729::supp*. **(A)** Among small bluish colonies, large dark blue-greenish colonies (indicated by arrows) appeared during cultivation of the Δ*sll0729* mutant on plates. **(B)** Growth as increase in OD_730_ of WT and Δ*sll0729::supp*_1 during cultivation under high CO_2_, 50 μmol photons m^–2^ s^–1^ PAR. Cells were inoculated at an OD_730_ of 0.15 at day 0. **(C)** Absorption spectra to show overall pigmentation of WT and Δ*sll0729::supp*_1. **(D)** Appearance of liquid cultures of the WT, the two suppressor strains (Δ*sll0729::supp*_1, Δ*sll0729::supp*_15), and the original mutant Δ*sll0729*, respectively. We show typical results from a representative experiment, which were reproduced in several independent experiments.

During the repeated cultivation of cells for the expression experiments, we noticed a gradual loss of the pigmentation phenotype that was initially observed for mutant Δ*sll0729*. Therefore, we spread diluted mutant cell suspensions on agar plates containing kanamycin. After 1 week two colony types appeared (Figure 2A). The majority of colonies was small and bluish as found for the initial mutant Δ*sll0729*, whereas also larger colonies appeared with a pigmentation similar to the WT. The divergence into these two colony types was reproducibly observed during independent experiments. The frequency of WT-like colonies increased over time when mutant Δ*sll0729* suspensions were cultivated for several weeks in liquid media. Retransformation of the original construct to obtain mutant Δ*sll0729* also resulted in non-stable mutant phenotypes, i.e., after few generations WT-like colonies appeared again. The clones Δ*sll0729::supp*_1 and Δ*sll0729::supp*_15 were further analyzed. They showed similar growth as the WT (Figure 2B) in liquid cultures under our standard conditions, consistent with their WT-like behavior on plates. Their pigmentation was also restored and similar to WT (Figures 2C,D).

**FIGURE 3:**
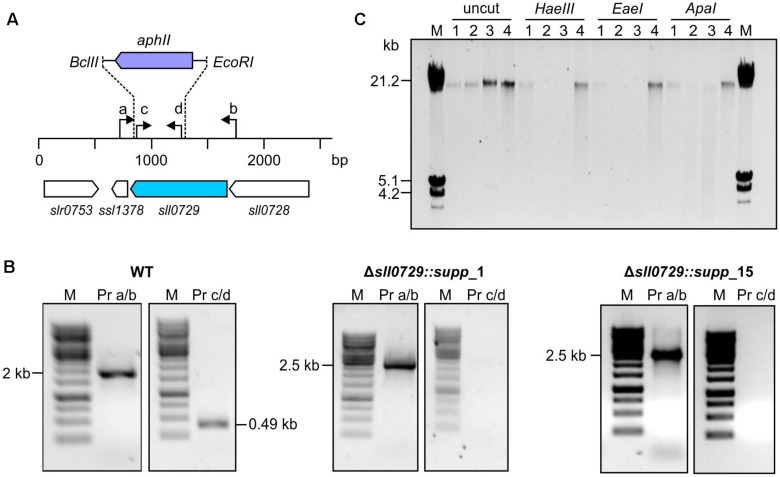
Genotypic analysis of *Synechocystis* WT and Δ*sll0729* suppressor clones. **(A)** Schematic view of *sll0729* mutation and primer-binding sites. **(B)** PCR with DNA of the WT and the suppressor mutants Δ*sll0729::supp*_1 and Δ*sll0729::supp*_15 using primer (Pr) combinations a/b and c/d, respectively. **(C)** Restriction analysis for functional verification of M.Ssp6803II encoded by *sll0729.* Chromosomal DNA of WT (1), Δ*sll0729::supp_*1 (2), Δ*sll0729::supp_*15 (3), and the Δ*sll0729+0729* complementation strain (4), respectively, was incubated with GGCC-specific, methylation-sensitive restriction endonucleases (*Hae*III, *Eae*I, and *Apa*I). DNA fragment patterns were analyzed using agarose gel electrophoresis.

To analyze this further, the genotype of WT-like colonies was investigated to clarify whether the mutation of *sll0729* was reverted into the original WT sequence or if suppressor mutations restored the phenotype. Via PCR we confirmed that the *sll0729* gene was still disrupted in these clones (Figure 3). By using primers, which bind outside of the deleted region of *sll0729*, an enlarged fragment was obtained when DNA of the suppressor mutant was used as PCR template, while a fragment that would be expected for a WT-like locus did not appear (Figure 3B). Due to the polyploidy of *Synechocystis*, we also had to confirm that the deleted sequence within the *sll0729* coding sequence was indeed absent in the Δ*sll0729* mutants. Therefore, primers that bind inside this deleted fragment were used for another PCR. Accordingly, in contrast to the WT, no PCR product was obtained for the mutants (Figure 3B). These results confirmed that the genotype of the Δ*sll0729* mutant was stable regardless of the instable phenotype, therefore, the clones growing like the WT represent suppressor mutants.

Finally, we also analyzed the M.Ssp6803II-specific methylation pattern of DNA in the isolated suppressor mutants. Total DNA was isolated from the WT, the two isolated suppressor mutants, and the Δ*sll0729* strain that was complemented by ectopic expression of *sll0729* (Δ*sll0729*+*sll0729*). The isolated DNA was treated with different restriction enzymes that are known to be inhibited by GG^m4^CC (Figure 3C). DNA isolated from the WT and the complementation strain Δ*sll0729*+*sll0729* resisted the action of *Hae*III, *Eae*I, or *Apa*I, whereas DNA of the two suppressor mutant clones could be cut with these enzymes as observed before for the original mutant Δ*sll0729* (Hagemann et al., 2018). Hence, corresponding to the genotype the activity of M.Ssp6803II was still completely abolished in the suppressor mutants.

### Physiological Characterization of the Suppressor Mutant Strains Δ*sll0729::supp*

**FIGURE 4:**
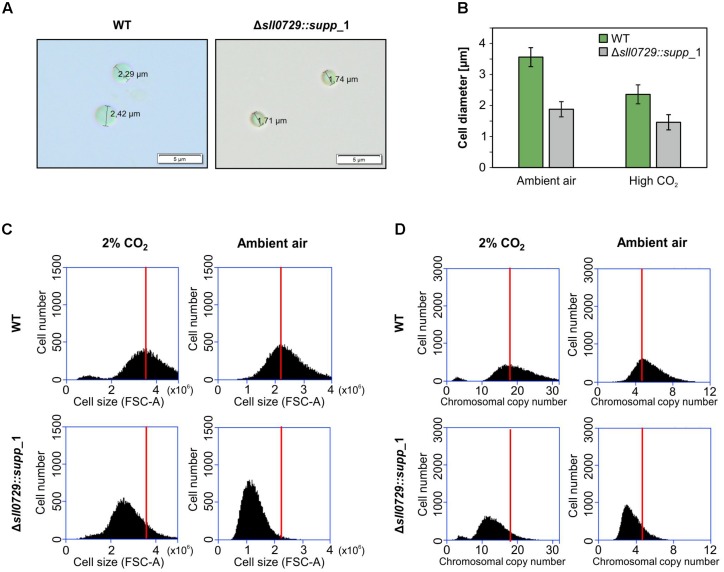
Cell size and DNA content of the suppressor mutant clones. **(A)** Microscopic observations of WT and Δ*sll0729::supp*_1. **(B)** Mean diameter of 100 cells of wild type (WT) and Δ*sll0729::supp*_1 after cultivation under ambient air and CO_2_-supplelemented conditions, respectively. **(C,D)** Cell size and chromosomal copy number profiles of suspensions from the WT or the Δ*sll0729::supp*_1 suppressor mutant measured by using FACS. Cells were grown under 2% CO_2_ or ambient air conditions for 24 h. 30,000 cells are analyzed using FACS. The distributions of cell size **(C)** and chromosome number **(D)**, estimated from FSC-A and FL1-H, respectively, are shown. The peaks of WT samples in each condition were indicated by red bars.

The suppressor clones permit the analysis of stable phenotypical alterations in strains with abolished GG^m4^CC DNA methylation. Despite similar appearance (Figure 2), light-microscopic inspections of WT and suppressor mutant cells showed that the cell diameter of suppressor mutant cells was approximately 50% smaller compared to the WT (Figures 4A,B). Moreover, this was the case at standard growth conditions as well as at higher growth rates, i.e., in presence of increased CO_2_ concentrations.

Subsequently, cell suspensions were analyzed regarding cell size and DNA contents using fluorescence-activated cell sorting (FACS). As observed in the microscope, the mean diameter of suppressor mutant cells was significantly smaller compared to the WT after growth of cells at ambient air or high CO_2_ conditions (Figure 4C). In both cases the mean cell diameter was reduced by 30–50%. Moreover, the smaller cells of the suppressor mutants also contained significantly less DNA than the WT (Figure 4D) regardless if grown under CO_2_-supplemented or ambient air conditions. Air-grown cells had always relatively lower DNA contents than cells from CO_2_-supplemented cultures. This change was found for WT cells as well as cells of the Δ*sll0729::supp*_1 strain (Figure 4D). In addition to cells obtained from exponential growth after 24 h, we also analyzed cells from other time points. Mutant cells showed always smaller cell size and contained less DNA compared to the WT (data not shown).

**FIGURE 5:**
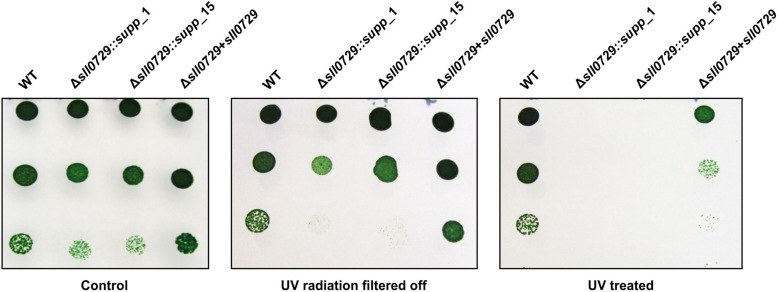
Comparison of the UV tolerance of wild type (WT), the two suppressor mutant strains (Δ*sll0729::supp_*1, Δ*sll0729::supp_*15) and the complementation strain (Δ*sll0729*+*sll0729*). Left, Control plate with non-treated cells. Middle, Control plate with cells covered by a 400 nm cut-off filter foil (Folex PR, Folex, Dreieich, Germany) resulting in UV-A and UV-B elimination. Right, Plate with cells exposed to UV-light UV-A 9.5 W m^–2^, UV-B 0.6 W m^–2^ in the presence of white light (PAR 75 μmol photons m^–2^ s^–1^) for 36 h. Photographs of a typical drop-dilution-assay are shown. After incubation, 5 μl of diluted cell suspension (1:10, 1:100, or 1:1000) were dropped on BG11 agar plates without antibiotics and incubated under standard conditions for 5 days. We show typical results, which were reproduced in three independent experiments.

In addition to the standard conditions, the growth of the suppressor mutants and the WT were compared under different light intensities (50, 75, 100, or 200 μmol photons m^–2^ s^–1^) when aerated with ambient air or 5% CO_2_ supplementation. The suppressor clones grew like WT under these conditions. It has been proposed that DNA methylation could play a role in specific DNA repair processes among cyanobacteria (Elhai, 2015). Therefore, we analyzed the resistance to UV in WT, suppressor mutants, and the complementation strain. Cells were exposed to UV-A 9.5 W m^–2^, UV-B 0.6 W m^–2^, and PAR 75 photons m^–2^ s^–1^ for 36 h. Cells of the WT and the complemented strain resisted the UV treatment, whereas cells of the suppressor mutants were sensitive to this treatment. Under non-stress conditions or when shielded against the UV part of the irradiation, all strains remained viable (Figure 5). Hence, *Synechocystis* cells lacking GG^m4^CC methylation became significantly more sensitive to UV radiation compared to the WT and the complementation strain.

### Methylation of GGCC Motifs Overlapping the -35 Region Influences Promoter Activity

**FIGURE 6:**
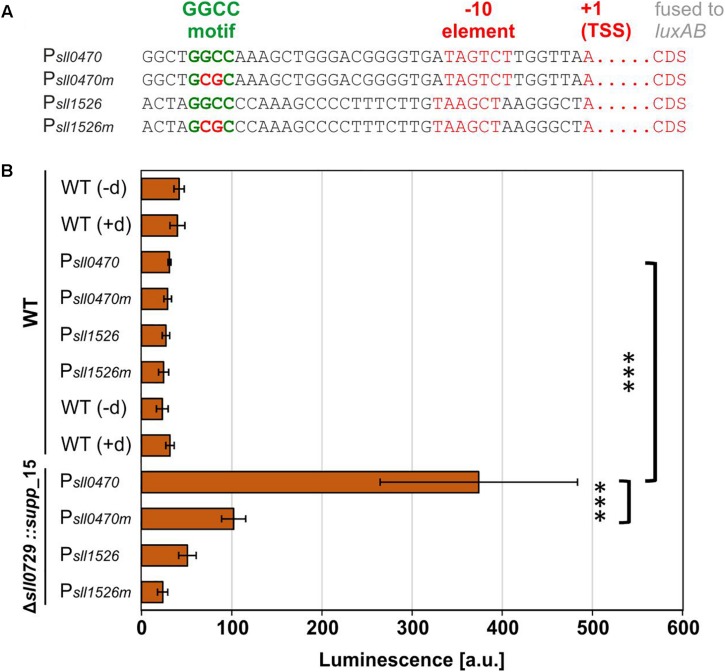
Promoter test experiments in WT or suppressor mutant background. Promoter activities of *sll1526* and *sll0470* were measured in *Synechocystis* WT or Δ*sll0729::supp_*15 *luxAB* reporter strains. **(A)** Promoter regions of *sll1526* and *sll0470* possess upstream of the transcriptional start site (TSS, +1) a GGCC motif. The information about the TSS was taken from Kopf et al. (2014). CDS, coding sequence. **(B)** Luciferase-mediated bioluminescence of P*_sll0470_* or P*_sll1526_ luxAB* fusions in the WT or in the Δ*sll0729::supp_*15 after incubation with decanal. The GGCC motif was changed to GCGC in the promoters suffixed by “m” (P*_sll0470m_* and P*_sll1526m_*). The WT without *luxAB* genes was used as negative control in the presence (+d) and absence (−d) of decanal. All measurements were performed in biological quadruplicates; the luminescence is given in arbitrary units (a.u.). Statistical analysis was performed using the two-tailed unpaired Student’s *t*-test (^∗∗∗^*p* < 0.001).

The genes *sll0470* and *sll1526* that are responding to the lack of methylation in the Δ*sll0729* mutant (Table 1) possess a GGCC motif, which is located in the promoter region upstream of the respective transcriptional start site (TSS). In both cases the motif overlaps with the region around −35 (referred to the TSS, +1) that is often crucial for promoter activity in bacteria (Figure 6A). The lacking methylation in the suppressor clones provided an opportunity to test, if the GGCC motif and the prevailing or missing methylation were directly responsible for the observed expression changes in a background more phenotypically similar to WT. Hence, the promoter activities of both genes were tested in reporter gene assays. For this purpose, promoter regions of *sll1526* and *sll0470* were transcriptionally fused to the luciferase-encoding *luxAB* genes. In each case, we inserted the WT promoter sequence with intact GGCC and a version with point-mutated sites (GGCC changed to GCGC) upstream of the reporter gene. The constructs were put into a neutral site on the chromosome of WT and Δ*sll0729::supp_*15 mutant cells. Confirmed clones were grown under standard growth conditions and luciferase activity was measured after addition of decanal. In WT cells only very low promoter activities, not significantly different from background activities, were found (Figure 6B). However, P_sll0470_ showed significantly increased activity in the background of mutant Δ*sll0729::supp_*15, which was reduced by about 75% when the mutated promoter version was tested (Figure 6B). These results indicate that methylation of the GGCC motif in P_sll0470_ represses its activity, since only in the Δ*sll0729::supp_*15 mutant background promoter activity was measured. Moreover, the GGCC motif upstream of *sll0470* plays an important role for the promoter strength, since mutation of GGCC resulted in significantly lowered promoter activity. Unfortunately, activity of the P_sll1526_ was below detection limit, i.e., its bioluminescence was not significantly different from the WT control, in the background of WT as well as of Δ*sll0729::supp_*15 (Figure 6B).

To improve promoter activity detection, we checked the expression pattern of these genes under different environmental conditions and found that the gene *sll1526* is induced under cold stress conditions (Kopf et al., 2014). Therefore, we repeated the promoter activity measurements with cold-stressed cells. Unfortunately, the activity of P_sll1526_ remained below threshold of the luminescence assay, whereas the P_sll0470_ was stimulated and showed higher activity in the mutant Δ*sll0729::supp_*15 background, which became again lowered by 75% with the mutated version as observed before under standard conditions (results not shown). Altogether, the results show the functional relevance of the GGCC motif within P_sll0470_.

### Proteome Analysis of the Suppressor Strains Δ*sll0729::supp* Lacking GG^m4^CC Methylation

The stable suppressor mutants were also used to analyze the impact of missing GG^m4^CC methylation on gene expression in *Synechocystis*. Here, we used protein extracts from three biological replicates for gel-free proteomics. To improve protein detection, in addition to the total protein extract a membrane-enriched fraction was analyzed. The approach allowed the relative quantification of 1707 proteins, which had been identified by at least two unique peptides. Thus, 48.7% of the total cellular proteome of *Synechocystis* could be investigated (see our PRIDE submission for the complete proteome data set). Among them, only 13 proteins showed significant changes in the two investigated suppressor mutant strains, Δ*sll0729::supp_*1 or Δ*sll0729::supp_*15, compared to all other investigated strains. Interestingly, the Sll1526 protein was identified as showing also decreased protein levels in the two suppressor strains (Table 2), matching the finding of its decreased mRNA level in the original Δ*sll0729* mutant. The protein Sll1527, which is encoded downstream from Sll1526, probably in the same operon, showed also decreased protein abundances. This finding is consistent with a slightly decreased mRNA abundance, which was also observed in the original Δ*sll0729* mutant as described above. The Sll0470 protein was also detected in the proteome data set. But it showed only 1.5 fold increase in the membrane fractions of the two suppressor mutant strains, which is below our threshold in proteomics, whereas it was significantly upregulated at mRNA level in Δ*sll0729::supp_*1 (log_2_ FC = 0.61, *P* < 0.05) and Δ*sll0729::supp_*15 (log_2_ FC = 1.11, *P* < 0.05).

**TABLE 2:** Proteome analysis of protein extracts from cells of the wild type and the suppressor mutants.

			**Membrane-enriched**		**Total extract**	
**Acc. ^A^**	**Acc. ^B^**	**Description**	**Δ*sll0729::supp*_1/WT**	**Δ*sll0729::supp*_15/WT**	**Δ*sll0729::supp*_1/WT**	**Δ*sll0729::supp*_15/WT**
P73077	Sll1941	DNA topoisomerase 4 subunit A, ParC	–1.84	–2.05	n.d.	n.d.
P73646	Sll1760	Homoserine kinase, ThrB	–1.08	–1.19	n.d.	n.d.
P73655	Slr1844	Tryptophan–tRNA ligase, TrpS	–1.12	–1.05	–1.16	–1.11
P73956	Sll1414	Protein Thf1, Psb29	–1.09	–1.03	–1.03	–1.19
P74359	Sll1527	Putative glycosyltransferase (GT4 family)	–1.49	–1.26	n.d.	n.d.
P74360	Sll1526	Methyltransferase domain-containing protein of unknown function	–1.11	–0.99	–1.65	–1.50
Q55612	Slr0776	UDP-3-O-acylglucosamine N-acyltransferase, LpxD	–1.45	–1.24	–1.49	–1.30
P74302	Slr0937	Chromosome segregation protein SMC homolog	n.d.	n.d.	1.07	1.41
P74332	Slr0959	Hypothetical protein	1.05	0.92	n.d.	n.d.
Q55982	Slr0660	4-hydroxythreonine-4-phosphate dehydrogenase, PdxA	0.99	1.01	n.d.	n.d.
Q6YRQ7	Slr6097	Type I site-specific deoxyribonuclease	1.15	1.24	n.d.	n.d.
Q6YRQ8	Slr6096	Type I restriction-modification system, M subunit	1.21	1.59	1.62	1.91
Q6YRQ9	Slr6095	Type I restriction-modification system, M subunit, M.Ssp6803V	0.99	1.07	1.47	1.57

*Cells of the WT and the clones Δsll0729::supp_1 or Δsll0729::supp_15 were grown under standard conditions. Total protein extracts and membrane-enriched fraction were analyzed. Mean values of proteome analyses from three biological replicates are given as log_2_ fold changes (FCs) compared to WT and fulfill the set significance criteria, namely a log_2_ FC > 1 or < −1 and a corresponding P-value < 0.01. A: Uniprot accession numbers. B: CyanoBase accession numbers. n.d. – not detected.*

However, the most remarkable change was found for Sll1941, the DNA topoisomerase 4 subunit A, which was reduced about fourfold in the membrane fraction of the suppressor mutants compared to WT (Table 2). The UDP-3-O-acylglucosamine N-acyltransferase (LpxD, Slr0776), which is linked to cell wall biosynthesis, also showed clearly reduced levels in total protein extracts as well as in the membrane fraction. Furthermore, the homoserine kinase (ThrB, Sll1760), tryptophan-tRNA ligase (TrpS, Slr1844), and the Thf1 protein, which was identified as photosystem II subunit Psb29 (Keren et al., 2005), were detected with decreased protein amounts in the suppressor mutant strains. We found only six proteins showing higher protein levels in the suppressor mutants compared to WT. Interestingly, among the up-regulated proteins, we found three proteins encoded on plasmid pSYSX, which are annotated to form a type 1 restriction modification system in *Synechocystis* (CyanoBase). Among them, Slr6095 has recently been identified as DNA methyltransferase M.Ssp6803V, which is probably responsible for the methylation of the bipartite motif GG^m6^AN_7_TTGG/CCA^m6^AN_7_TCC (Hagemann et al., 2018). In addition, the amounts of the 4-hydroxythreonine-4-phosphate dehydrogenase (PdxA, Slr0660), the chromosome segregation protein SMC homolog (Slr0937), and of one protein of unknown function (Slr0959) were found at elevated levels in our proteomic data set (Table 2).

**FIGURE 7:**
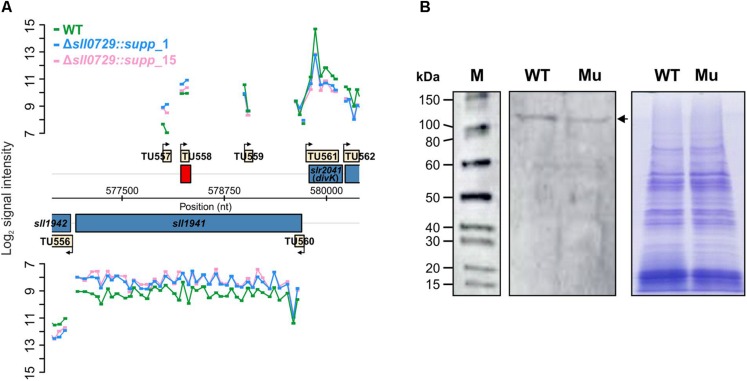
Verification of diminished expression of the DNA topoisomerase 4 (Sll1941) in the suppressor mutant. **(A)** Signal intensities of probes covering the *sll1941* gene on the *Synechocystis* chromosome are shown, which visualize the transcriptional changes of *sll1941* in the microarray experiment. For this microarray experiment we used two biological and three technical replicates. **(B)** The decreased protein abundance of Sll1941 was verified by a Western-blot experiment. In each lane 7.5 μg of extracted total protein were loaded. The left panel shows the chemiluminescence-based detection of the Sll1941 protein (marked by an arrow) via a specific antibody. The right panel shows the Coomassie-stained gel to verify equal protein loading. (M – protein size marker, WT – wild type, Mu – Δ*sll0729::supp*_1).

The decreased abundance of Sll1941, the DNA topoisomerase 4 subunit A was verified by Western-blot experiments (Figure 7). Using an antibody specific for Sll1941 we detected a lowered signal at the expected size of approximately 101 kDa in extracts of the mutant Δ*sll0729::supp*_1 compared to WT, corresponding to the lowered abundance of this protein in the proteome data set. Furthermore, the DNA topoisomerase 4 expression was also checked on mRNA level. Corresponding with proteomics, we found a decreased abundance of *sll1941* transcripts in the suppressor mutant clones Δ*sll0729::supp*_1 (log_2_ FC = −0.87, *P* < 0.05) or Δ*sll0729::supp*_15 (log_2_ FC = −1.00, *P* < 0.05) compared to the WT (Figure 7).

## Discussion

*Synechocystis* encodes five different DNA-specific methyltransferases. Among these methyltransferases, the mutation of *sll0729* encoding M.Ssp6803II resulted in clear phenotypical alterations under conditions promoting high growth rates, i.e., the mutant Δ*sll0729* appeared bluish due to lowered chlorophyll *a* content and grew slowly compared to WT (Hagemann et al., 2018). These initial findings led to the hypothesis that the missing GGCC methylation, which occurs at high frequencies in the *Synechocystis* genome including many promoter regions, could have a global impact on the gene expression pattern resulting in these strong phenotypical alterations. To support this assumption, we performed transcriptomics. In contrast to our expectations, only a few genes showed significant changes in gene expression (Supplementary Table S2). Moreover, most of these differentially regulated genes lack GGCC sites within their promoter sequences suggesting that their de-regulation was caused by indirect effects. This assumption is supported by the finding that repeated transcriptome analyses (*n* = 4) always identified different sets of de-regulated genes in mutant Δ*sll0729*, with the exception of only two genes. The mRNAs of the genes *sll1526* and *sll0470* were reproducibly detected at lower and higher levels, respectively, in the mutant lacking M.Ssp6803II activity, which was supported by Northern-blotting experiments (see Figure 1). Comparable observations have recently been made in different *H. pylori* strains. The mutation of a DNA methyltransferase conserved in all known *H. pylori* genomes also resulted in the changed gene expression of several genes; but similar to our observations here, only 10 genes showed changes consistently (Estibariz et al., 2019).

The functions of the proteins encoded by *sll1526* and *sll0470* are unknown. However, the N-terminal part of Sll1526 contains a methyltransferase domain (pfam13649) and shows similarities to SmtA (COG0500) characteristic for S-adenosyl-methionine transferases, while the central protein part is related to the pfam10119, which is annotated as regulatory domain for prokaryotic methyltransferases. The occurrence of these protein domains make it likely that Sll1526 represents a methyltransferase with yet unknown substrate specificity in *Synechocystis*. BLAST-P searches revealed that proteins of high similarity are only present in some closely related cyanobacterial strains such as *Synechocystis* sp. PCC 6714, diverse *Microcystis* spp., *Cyanobacterium stranieri* PCC7202, *Gloeomargarita lithophora* and a few more distantly related cyanobacteria, and some proteobacteria. The restricted distribution of Sll1526 homologs among cyanobacteria make it unlikely to assume that the putative methyltransferase Sll1526 and the DNA-specific methyltransferase M.Ssp6803II are functionally cooperating, because homologs of M.Ssp6803II are found in numerous strains from almost all cyanobacterial clades (Hagemann et al., 2018). In the Sll0470 protein, a transmembrane domain is predicted from position 19 to 38, followed by a domain of unknown function (DUF2808), which is restricted to the cyanobacterial phylum. Consistently, BLAST-P searches showed that genes encoding proteins with high similarity to Sll0470 exist in numerous genomes from all cyanobacterial clades, where they are often located in the neighborhood of the *pntA* gene (see Supplementary Figure S1). The pyridine nucleotide transhydrogenase (PntA) is involved in the maintenance of proper NADH/NADPH ratios especially under photomixotrophic conditions (Kämäräinen et al., 2017). However, a functional connection between these two proteins is unclear at present.

The two genes *sll1526* and *sll0470*, which exhibited stable changes in mutant Δ*sll0729*, both contain a GGCC site overlapping the -35 element in their promoters. Using the Δ*sll0729::supp*_15 strain as host, the importance of GGCC and its methylation could be verified using promoter::*luxAB* gene fusions for P*_sll0470_*. Consistent with the increased transcript level of *sll0470* in the mutant Δ*sll0729* we found significantly higher reporter gene expression in the Δ*sll0729::supp*_15 background. The observed lowered reporter gene expression when using the *sll0470* promoter in which GGCC sites were changed to GCGC is best explained by the disturbance of the -35 element in the promoter, which had a negative impact on the overall promoter activity (see Figure 6). The promoter activities were measured in cells of the suppressor mutant. Therefore, we cannot completely rule out that these changes might be related to other issues than DNA methylation. However, we observed the similar stimulation of *sll0470* expression in the original Δ*sll0729* mutant and the suppressor mutant clone, therefore, we assume that most likely lacking DNA methylation was responsible for the stimulated *sll0470* expression. In case of *sll1526* the activity of the promoter was below the detection limit of the applied promoter test system. These experiments verified that GGCC methylation is relevant for promoter function at least in the case of *sll0470* and absent methylation results in overexpression of the *sll0470* gene.

Collectively, our transcriptome analyses pointed at specific rather than global MSsp6803II-dependent transcriptional changes, which is in strong contrast to the wide distribution of the GGCC motif occurring more than 38.000 times in the *Synechocystis* chromosome. We only found altered expression of the *sll0470* and *sll1526* genes with GGCC motifs overlapping the -35 promoter element. Due to the largely unknown function of their encoded proteins it is difficult to conclude that changed expression of these genes could be responsible for the strong pigmentation and growth phenotype of the initial Δ*sll0729* mutant. This assumption is supported by the later finding that these two genes remained deregulated in the suppressor mutant clones, which show pigmentation and growth similar to WT. Therefore, we conclude that the main meaning of GGCC methylation is different from (global) transcriptional regulation.

During our further experiments we noticed that the initially observed strong phenotypical alterations in mutant Δ*sll0729* decreased over time, which led to the identification of many suppressor colonies, which appeared spontaneously. A connection between DNA methylation and mutation rates has often been reported (e.g., Cherry, 2018). The frequent occurrence of suppressor mutants indicates that it is genetically easy to escape the need for this ^m4^C methylation by one or possibly also several different mutations. Nevertheless, the isolated suppressor mutants appeared interesting, because they subsequently permitted the analysis of GGCC-methylation-specific effects and revealed that the cells can display a WT-like phenotype also in the absence of M.Ssp6803II methylation. Thus, these findings supported the above mentioned conclusion that most of the strong phenotypical alterations reported for the initial mutant Δ*sll0729* could be rather indirectly connected to GGCC methylation.

The two investigated suppressor mutant clones showed similar changes, they formed smaller cells with lowered DNA content, which might indicate that these suppressor mutant cells are characterized by lower DNA copy number. A close correlation between DNA copy number and cell size has been reported for *Synechococcus elongatus* PCC 7942 (Zheng and O’Shea, 2017), whereas variable DNA copy numbers have been reported for *Synechocystis* under different growth conditions (Zerulla et al., 2016). The suppressor mutant clones are also less UV tolerant than WT cells. This effect could be related to the proposed role of DNA methylation for DNA repair processes as has been suggested especially for the HIP1-related methylation via M.Ssp6803I by Elhai (2015). The lowered UV tolerance could also result from assuming a lowered DNA copy number in the suppressor clones. High DNA copy numbers are known to support DNA repair processes and resistance toward harsh environmental stresses in bacteria such as *Deinococcus radiodurans* (reviewed in Lim et al., 2019). Collectively, these findings connect GGCC methylation with DNA stability, replication and repair.

The hypothetical role of GGCC methylation in DNA-related processes was also supported by the proteome analysis, which revealed a fourfold lower amount of the protein Sll1941 in the two suppressor mutant strains compared to WT (Table 2). Sll1941 is annotated as topoisomerase 4 subunit A (ParC) in the Uniprot database^1^, whereas the same protein is annotated as gyrase subunit A (GyrA) in CyanoBase^2^. Both topoisomerase 4 and gyrase impact DNA supercoiling; however, they fulfill distinct functions. DNA gyrases remove positive supercoils in an ATP-dependent reaction during transcription and replication, while ATP-dependent DNA topoisomerases relax negative supercoils and are specifically involved in chromosome partitioning (Dorman and Dorman, 2016). Therefore, it is important to correctly annotate Sll1941. The *Synechocystis* genome harbors the Slr0417 protein displaying 61% identical positions with Sll1941. Slr0417 is annotated as gyrase subunit A in Uniprot^3^ as well as in CyanoBase^4^. BLAST-P searches against amino acid sequences from model organisms revealed that Slr0417 displays higher similarities with DNA gyrase subunit A from *E. coli* and other model bacteria than Sll1941, pointing at the correct annotation of Slr0417 as GyrA in *Synechocystis*. Gyrase is composed of two subunits, GyrA and GyrB. The gene *sll2005* is annotated as *gyrB* in *Synechocystis*, and the two putative gyrase subunits Slr0417 and Sll2005 showed unchanged abundances in the proteome of suppressor mutants. Hence, it is likely that Sll1941 rather represents topoisomerase 4 subunit A than gyrase in *Synechocystis*.

In *E. coli* and related bacteria, DNA topoisomerase 4 is the principal decatenase, responsible for the topological separation of daughter chromosomes before cell separation (Dorman and Dorman, 2016). This function might be even more important in bacteria harboring a multi-copy genome such as *Synechocystis* (Zerulla et al., 2016). The presumable function of Sll1941 as *Synechocystis* topoisomerase 4 subunit A is consistent with the correlation between lowered DNA amounts and lowered topoisomerase 4 abundance in cells of the suppressor clones. Moreover, topoisomerase 4 is able to reduce the negative supercoiling of DNA similarly to DNA topoisomerase 1, which is important to sustain transcription and replication in bacteria (Dorman and Dorman, 2016). The *Synechocystis* topoisomerase 1 (TopA, Slr2058) showed similar protein amounts in the suppressor mutant and WT. Hence, it is possible to speculate that DNA methylation might also have an effect on supercoiling or DNA organization. Consistent with that idea we observed also a different accumulation of the Slr0937 protein that contains a chromosome segregation (TIGR02168) domain (Table 2). These observations point to the possibility that less-methylated DNA due to the abolished M.Ssp6803II activity might not be organized correctly, in turn leading to the rather strong phenotype initially observed in mutant Δ*sll0729*. Reduced expression of topoisomerase 4 and an assumed decrease in the overall activity might complement this methylation defect to a great extent leading to the less severe phenotype in the suppressor mutant cells. At present, we do not know how the reduced *sll1941* expression is achieved in the suppressor clones of *Synechocystis*. The sequence of the gene and its promoter is not changed in the suppressor mutants compared to WT. Interestingly, the *sll1941* promoter region also contains GGCC sites, which might have an impact on *sll1941* expression when not methylated. It has been shown in model bacteria that the degree of DNA supercoiling is giving feedback to the topoisomerase gene expression to maintain the superhelical density in the bacterial DNA (Snoep et al., 2002). Obviously, the reduced topoisomerase 4 expression that was found in the two suppressor strains could thus compensate the missing GGCC methylation on DNA structure.

Nevertheless, several of the other deregulated proteins could also be related to the observed changes in the suppressor strains. For example, the UDP-3-O-acylglucosamine N-acetyltransferase is involved in cell wall biogenesis, hence, its down-regulation correlates with the reduced cell size of the suppressor mutants. Interestingly, all proteins encoded by the *slr6095-97* operon on plasmid pSYSX were found in higher abundances in the suppressor clones. The protein Slr6095 represents M.Ssp6803V methylating a rather rare bipartite motif on the *Synechocystis* DNA (Hagemann et al., 2018). One might speculate that an increased methylation activity of this protein might be partially compensating the absence of GGCC methylation via M.Ssp6803II.

Collectively, these findings show that GGCC methylation can play multiple roles in *Synechocystis*. It is regulating gene expression of at least two genes, where GGCC sites are found near the -35 promoter elements. However, the impact on gene expression regulation via M.Ssp6803II was much lower than initially expected. In contrast, we received some strong indications that methylation of GGCC sites rather plays an important role for DNA supercoiling and stability, which clearly affects DNA replication and repair mechanisms. It should be noted that most of these results were obtained with suppressor mutant clones, which still are completely defective in M.Ssp6803II activity. However, we cannot completely rule out that some of the changes might be related to other mutations acquired by the suppressor clones. Hence, further work with the suppressor mutant strains will be necessary to elucidate the underlying mechanisms.

## Data Availability

The datasets generated for this study can be found in GEO database, GSE126285.

## Author Contributions

MH and WRH designed the study. KG, SK, SW, SM, and IS, performed the experiments. KG, SK, SW, SM, IS, WRH, and MH analyzed the data. MH wrote the manuscript with contributions from all authors.

## Conflict of Interest Statement

The authors declare that the research was conducted in the absence of any commercial or financial relationships that could be construed as a potential conflict of interest.

## Footnotes

1 https://www.uniprot.org/uniprot/P73077

2 http://dev-genome.annotation.jp/cyanobase/GCA_000009725.1/genes/sll1941

3 https://www.uniprot.org/uniprot/Q55738

4 http://dev-genome.annotation.jp/cyanobase/GCA_000009725.1/genes/slr0417
