# Supplementary material for: Cytosine N4-Methylation via M.Ssp6803II Is Involved in the Regulation of Transcription, Fine- Tuning of DNA Replication and DNA Repair in the Cyanobacterium Synechocystis sp. PCC 6803
Source: Front Microbiol. 2019 Jun 5;10:1233. doi: 10.3389/fmicb.2019.01233 (PMC6560206; doi:10.3389/fmicb.2019.01233)
Supplement: Supplementary file 1 [file Data_Sheet_1.PDF]

**Supplementary material**

**Detailed description pf proteomic methods**

*In-solution digestion of proteins*

Cells were disrupted using Precellys 24 homogenizer (peqLab Biotechnologie GmbH, Erlangen, Germany) in a buffer containing 10 mM Tris/HCl, pH 7.4, 138 mM NaCl; 2.7 mM KCl; 1 mM $MgCl_2$. An aliquot of the resulting total protein extract was centrifuged at 22000 g for 100 min to obtain the membrane-enriched fraction.

Samples containing 75 µg protein in 100 µl extraction buffer (50 mM ammonium bicarbonate (ABC), 1.5% sodium deoxycholate (SDC), 10 mM dithiothreitol) were incubated at 95 °C for 5 min and subsequently sonicated for 10 min using a bath sonicator. Alkylation was performed with 15 mM iodoacetamide for 20 min at room temperature. Thereafter 50 mM ABC and sequencing grade trypsin (Promega) were added to obtain an enzyme/protein ratio of 1:50 in a final volume of 320 µl. Digestion was performed at 37 °C for about 16 h. SDC was removed from the digest solutions using the phase transfer surfactant method (Masuda et al. 2008). 320 µl of ethylacetate and 6.5 µl of 25% trifluoroacetic acid were added, the mixture was rigorously shaken for 3 min and subsequently centrifuged at 12000 g for 10 min to obtain aqueous and organic phases. 200 µl of the aqueous phase was collected using a gel loading tip. Finally, the peptide solutions were desalted with OASIS HLB 1cc Vac Cartridges (Waters, Manchester, UK).

*Analysis by nanoLC-HDMS$^E$*

LC-HDMS$^E$ analyses were carried out using a nanoAcquity UPLC system (Waters) coupled to a Waters Synapt G2-S mass spectrometer via a NanoLockSpray ion source. Mobile phase A contained 0.1% formic acid in water, and mobile phase B contained 0.1% formic acid in acetonitrile. Peptide samples corresponding to approximately 180 ng and 360 ng of digested protein from total extract and membrane-enriched fraction, respectively, supplemented with 40 fmol of Hi3 Phos B standard for protein absolute quantification (Waters) were trapped and desalted using a precolumn (nanoAcquity UPLC Symmetry C18, 5 µm, 180 µm x 20 mm, Waters) at a flow rate of 10 µl/min for 4 min with 99.9% A. Peptides were separated on an analytical column (ACQUITY UPLC HSS T3, 1.8 µm, 75 µm x 250 mm, Waters) at a flow rate of 300 nl/min using a gradient from 3% to 32% B over 150 min. The column temperature was maintained at 35 °C. The SYNAPT G2-S instrument was operated in data-independent mode with ion-mobility separation as an additional dimension of separation (referred to as HDMS$^E$). By executing alternate scans at low and elevated collision energy (CE) of each 0.6 sec, information on precursor and fragment ions, respectively, was acquired. In low-energy MS mode acquisitions were performed at constant CE of 4 eV whereas drift time-dependent CE settings (Distler et al. 2014) were applied in elevated-energy MS mode. As a reference

compound, 100 fmol/µl [Glu1]-fibrinopeptide B was delivered at 500 nl/min to the reference sprayer of the NanoLockSpray source. Lock spray was acquired once every 30 s for a 1 s period. Samples were measured once without technical replication.

*nanoLC-HDMS$^E$ data processing, protein identification and quantification*

Progenesis QI for Proteomics version 4.1 (Nonlinear Dynamics, Newcastle upon Tyne, UK) was used for raw data processing, protein identification and label free quantification. For Apex3D processing, the thresholds for low and high energy scan ions were set to 135 and 30 counts, respectively. Alignment was performed to compensate for between-run variation in the LC separation. Peak picking parameters included (i) sensitivity set automatic, (ii) maximum ion charge of +4. For the database search a database containing 3507 protein sequences from *Synechocystis* sp. (strain PCC 6803 / Kazusa) (UniProt release 2019_01) appended with the sequences of rabbit phosphorylase B (P00489) and porcine trypsin was compiled. Precursor and fragment ion mass tolerances were automatically determined. Two missing cleavage sites were allowed, oxidation of methionine residues was considered as variable modification, and carbamidomethylation of cysteines as fixed modification. The false discovery rate was set to 4%. Peptides were required to be identified by at least three fragment ions and proteins by at least six fragment ions. Subsequently peptide ion data were filtered to retain only peptide ions that met the following criteria: (i) identified at least two times within the dataset, (ii) ion score greater or equal 5.3 and 5.5 for the analysis of the total extract and the membrane-enriched fraction, respectively, (iii) mass error below 10.0 ppm or, exceptionally, below 13.0 ppm if the peptide was identified at least 8 times, and (iiii) at least 6 amino acid residues in length. Moreover, identification results based on charge state deconvolution were removed. Proteins were quantified by the absolute quantification Hi3 method using Hi3 Phos B Standard (Waters) as reference (Silva et al. 2006). Protein abundance changes by a factor of at least two, accompanied by ANOVA $p$-values < 0.01 were regarded as significant. Only proteins identified by at least two unique peptides were included in the quantitative analysis. To estimate the final rate of false peptide identifications, the search was repeated using a shuffled target-decoy database applying identical peptide and protein filtering criteria. Comparing the number of decoy peptides to those identified with the target sequences resulted in false positive rates below 0.1 %.

**References**

**Distler U, Kuharev J, Navarro P, et al.** 2014. Drift time-specific collision energies enable deep-coverage data-independent acquisition proteomics. Nature Methods **11,** 167-170.

**Masuda T, Tomita M, Ishihama Y.** 2008. Phase transfer surfactant-aided trypsin digestion for membrane proteome analysis. Journal of Proteome Research **7,** 731-740.

**Silva JC, Gorenstein MV, Li GZ, Vissers JP, Geromanos SJ.** 2006. Absolute quantification of proteins by LCMSE: a virtue of parallel MS acquisition. Molecular and Cellular Proteomics **5,** 144-156.

**Supplementary Table S1: Primers used in the present study.**

| Primer in Fig. 4 | Description | Sequence (5' - 3') | Binding sites |
|---|---|---|---|
| a | 0729DEL_fw | ATGATCTAGCTCCATGGCGA | Downstream of coding sequence of *sll0729* |
| b | 0729DEL_re | AGGTTATGACGATCCGGCTT | Upstream of coding sequence of *sll0729* |
| c | 0729Inside_fw | TTACTTTTTCTGGCACGGTG | Inside the deleted *sll0729* fragment |
| d | 0729Inside_re | GGAAGCTTTGTCTTGTTTGT | Inside the deleted *sll0729* fragment |

**Supplementary Table S2: Genes up- or down-regulated in Δ*sll0729* compared to WT and the corresponding values for the complementation strains Δ*sll0729*+*sll0729* and Δ*sll0729*+*ssl1378*.** Values are given as $\log_2$ fold changes (FC) compared to WT and were regarded as significant if > 1 or < -1 with a corresponding *P* value < 0.05. Significant FC are given in bold.

| Gene ID | TU | Δ*sll0729* | Δ*sll0729*+*sll0729* | Δ*sll0729*+*ssl1378* | Gene name | Description |
|---|---|---|---|---|---|---|
| slr0513 | TU3085 | **1.93** | **2.55** | 0.92 | NA | iron transport system substrate-binding protein, periplasmic protein |
| slr1295 | TU288 | **1.56** | **2.11** | 0.70 | sufA | iron transport system substrate-binding protein |
| ssr2333 | TU689 | **1.36** | **2.24** | 0.23 | NA | unknown protein |
| sll0470 | TU3100 | **1.30** | 0.12 | **1.30** | NA | hypothetical protein |
| sll1549 | TU3631 | **1.02** | **2.53** | 0.35 | NA | salt-enhanced periplasmic protein |
| sll1878 | TU1867 | **1.02** | **1.20** | 0.35 | NA | iron(III)-transport ATP-binding protein |
| sll1526 | TU2137 | **-1.05** | -0.08 | **-1.22** | NA | hypothetical protein |

**Supplementary Figure S1: Gene neighborhood of *sll0470* homologs (red arrow) in cyanobacterial genomes.** We used the SyntTax webserver (http://archaea.u-psud.fr/synttax/) and the Sll0470 amino acid sequence from *Synechocystis* sp. PCC 6803 as query to analyze synteny within the cyanobacterial phylum.

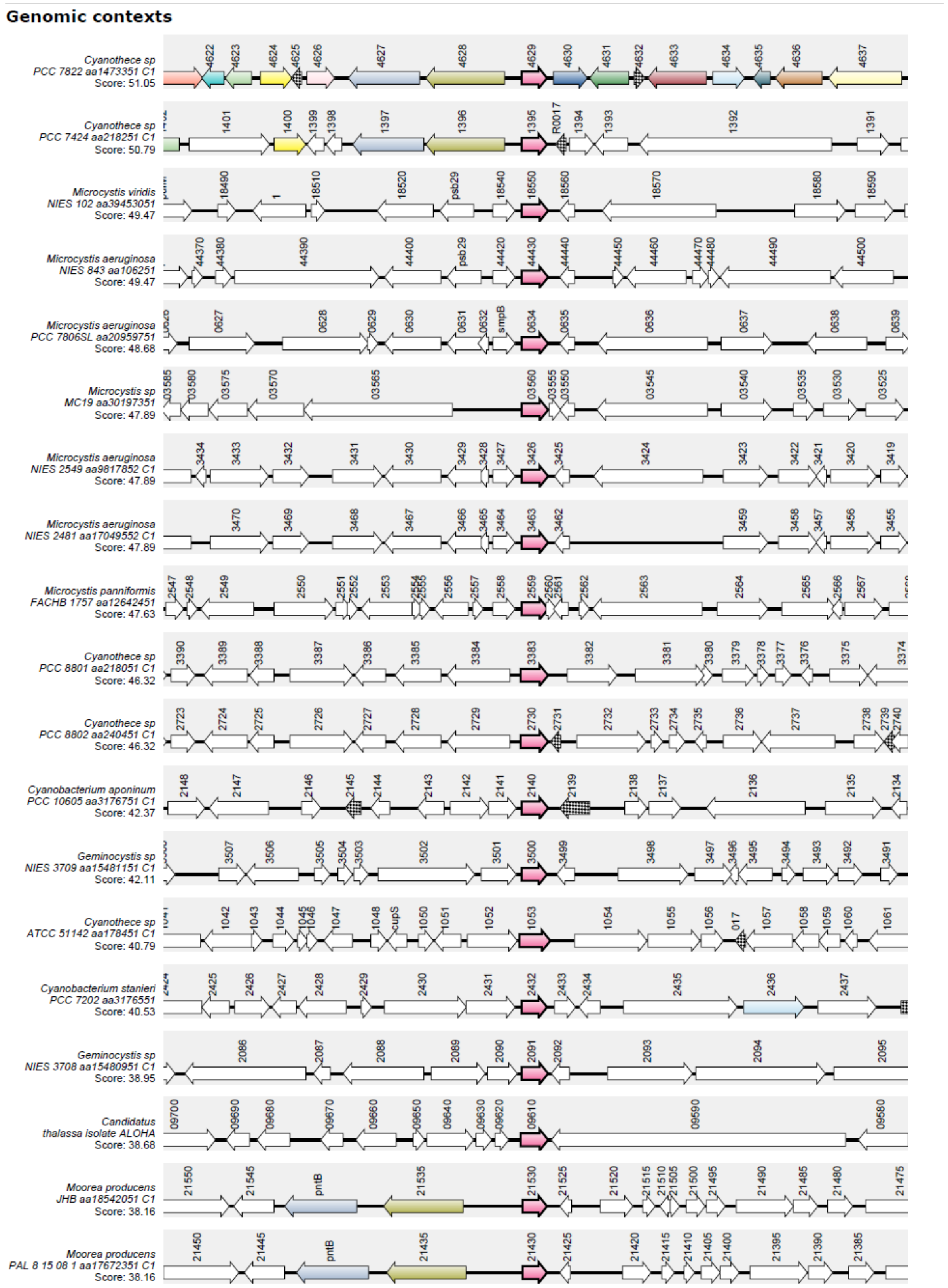

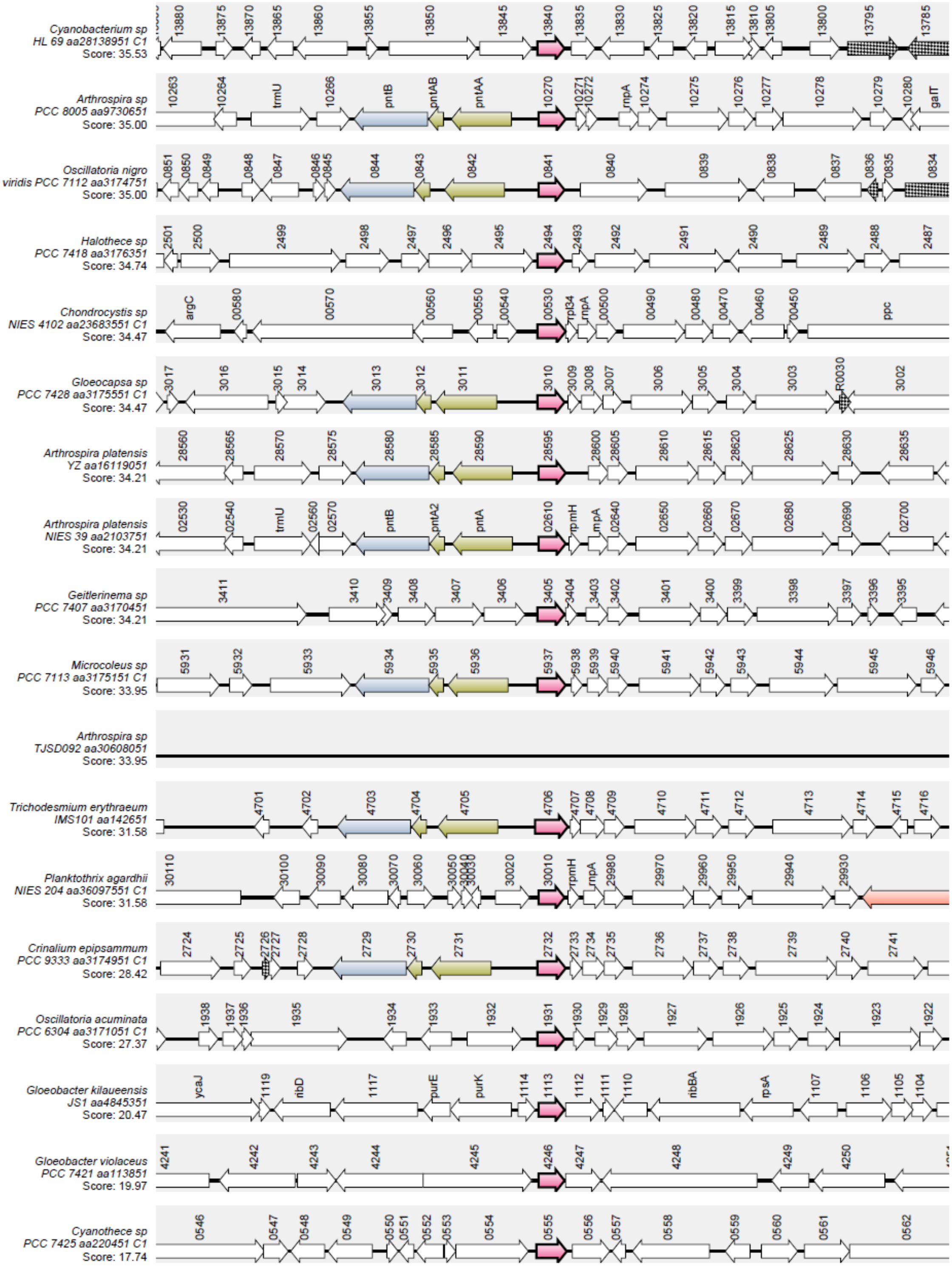

Cyanobacterium sp HL 69 aa28138951 C1 Score: 35.53
13880 13875 13870 13865 13860 13855 13850 13845 13840 13835 13830 13825 13820 13815 13810 13805 13800 13795 13785
Arthrospira sp PCC 8005 aa9730651 Score: 35.00
10263 10264 trmU 10266 pntB pntAB pntAA 10270 10271 10272 rnpA 10274 10275 10276 10277 10278 10279 10280 galT
Oscillatoria nigro viridis PCC 7112 aa3174751 Score: 35.00
0851 0850 0849 0848 0847 0846 0845 0844 0843 0842 0841 0840 0839 0838 0837 0836 0835 0834
Halothece sp PCC 7418 aa3176351 Score: 34.74
2501 2500 2499 2498 2497 2496 2495 2494 2493 2492 2491 2490 2489 2488 2487
Chondrocystis sp NIES 4102 aa23683551 C1 Score: 34.47
argC 00580 00570 00560 00550 00540 00530 rpl34 rnpA 00500 00490 00480 00470 00460 00450 ppc
Gloeocapsa sp PCC 7428 aa3175551 C1 Score: 34.47
3017 3016 3015 3014 3013 3012 3011 3010 3009 3008 3007 3006 3005 3004 3003 R0030 3002
Arthrospira platensis YZ aa16119051 Score: 34.21
28560 28565 28570 28575 28580 28585 28590 28595 28600 28605 28610 28615 28620 28625 28630 28635
Arthrospira platensis NIES 39 aa2103751 Score: 34.21
02530 02540 trmU 02560 02570 pntB pntA2 pntA 02610 rpmH rnpA 02640 02650 02660 02670 02680 02690 02700
Geitlerinema sp PCC 7407 aa3170451 Score: 34.21
3411 3410 3409 3408 3407 3406 3405 3404 3403 3402 3401 3400 3399 3398 3397 3396 3395
Microcoleus sp PCC 7113 aa3175151 C1 Score: 33.95
5931 5932 5933 5934 5935 5936 5937 5938 5939 5940 5941 5942 5943 5944 5945 5946
Arthrospira sp TJSD092 aa30608051 Score: 33.95
Trichodesmium erythraeum IMS101 aa142651 Score: 31.58
4701 4702 4703 4704 4705 4706 4707 4708 4709 4710 4711 4712 4713 4714 4715 4716
Planktothrix agardhii NIES 204 aa36097551 C1 Score: 31.58
30110 30100 30090 30080 30070 30060 30050 30010 rpmH rnpA 29980 29970 29960 29950 29940 29930
Crinalium epipsammum PCC 9333 aa3174951 C1 Score: 28.42
2724 2725 2726 2727 2728 2729 2730 2731 2732 2733 2734 2735 2736 2737 2738 2739 2740 2741
Oscillatoria acuminata PCC 6304 aa3171051 C1 Score: 27.37
1938 1937 1936 1935 1934 1933 1932 1931 1930 1929 1928 1927 1926 1925 1924 1923 1922
Gloeobacter kilaueensis JS1 aa4845351 Score: 20.47
ycaJ 1119 ribD 1117 purE purK 1114 1113 1112 1111 1110 ribBA rpsA 1107 1106 1105 1104
Gloeobacter violaceus PCC 7421 aa113851 Score: 19.97
4241 4242 4243 4244 4245 4246 4247 4248 4249 4250
Cyanothece sp PCC 7425 aa220451 C1 Score: 17.74
0546 0547 0548 0549 0550 0551 0552 0553 0554 0555 0556 0557 0558 0559 0560 0561 0562
